# EML1/402: An Electronic Mailing List for Radiologists: First year's experience

**DOI:** 10.2196/jmir.1.suppl1.e31

**Published:** 1999-09-19

**Authors:** E Ranschaert, S Achenbach

**Affiliations:** ^1^AZ Fusieziekenhuis H. Hart, NinoveBelgium

**Keywords:** Electronic Mail, Internet, Radiology, Medical Informatics

## Abstract

**Introduction:**

Radiologists can use the Internet to seek medical information, but also to distribute messages and images. The EUFORA mailing list was founded in July 1998 with the purpose to create an international electronic forum for radiologists. It was intended to be a list for discussion and collaboration as well as to be an information platform. An international team of 'co-owners' including several academics was established for continuous evaluation of the list, to ensure the quality of the postings. The Listbot list server has been used for running EUFORA. All messages were automatically archived and searchable per date, title and author. Subscriptions were made via the supporting web site (http://www.devolder.be/eufora). Every new candidate would get a voluntary sign-up form for registration. Access was restricted to medical doctors. The purpose of this study was to evaluate the activity on the mailing list, the types of subjects that are being discussed, and the participation degree of its members.

**Methods:**

Eleven consecutive calendar months from July 1998 to May 1999 were chosen for the evaluation. All messages were counted and classified into four categories. Messages concerning the handling of the list were put in the "administrative" category. The "case studies" group contained case quizzes and all mailings about radiology cases. Messages with an informative purpose, mainly announcements off congresses, were classified as "informative". The "general radiology" group consisted of all messages related to various aspects of radiology, and that could not be categorised otherwise. The number of contributors and the total number of replies was recorded in every month. Information from the voluntary sign-up form was used to obtain information about nationality, gender and age.

**Results:**

Over this eleven-month period the number of subscribers grew from 28 to 186, coming from 33 countries. A total of 260 messages has been sent to the list. Most of the messages could be categorised under "general radiology" (32%). 22% were case studies and 12% were purely informative. In total 76 replies were counted, 29% of all the messages that have been sent. Only 7% of all the subscribers made contributions to the list.

**Discussion:**

The increasing number of subscribers to EUFORA suggests that there is a growing interest for mailing lists, although one cannot exclude the increasing availability of the Internet as a cause for this expansion. The high percentage of replies shows that there is a good interaction, although the majority of participants is not contributing actively. It was impossible however to count the replies that had only been sent to the original authors, making it difficult to evaluate the real participation degree of all the subscribers. In conclusion we think that all the members experience this forum positively.

